# Prognostic value of the expression of C-Chemokine Receptor 6 and 7 and their ligands in non-metastatic breast cancer

**DOI:** 10.1186/1471-2407-11-213

**Published:** 2011-05-30

**Authors:** Philippe A Cassier, Isabelle Treilleux, Thomas Bachelot, Isabelle Ray-Coquard, Nathalie Bendriss-Vermare, Christine Ménétrier-Caux, Olivier Trédan, Sophie Goddard-Léon, Jean-Jacques Pin, Hervé Mignotte, Clarisse Bathélémy-Dubois, Christophe Caux, Serge Lebecque, Jean-Yves Blay

**Affiliations:** 1 Departments of Medecine, Centre Léon Bérard, Lyon, France; 2 Equipe Cytokine et Cancer, INSERM U590, Centre Léon Bérard, Lyon, France; 3 Departments of Pathology, Centre Léon Bérard, Lyon, France; 4 Université Claude Bernard Lyon 1, Lyon, France; 5 Departments of Surgery, Centre Léon Bérard, Lyon, France; 6 Innate Pharma, Marseille, France

**Keywords:** early breast cancer, chemokine, chemokine receptor, prognosis, metastasis

## Abstract

**Background:**

Chemokines and chemokine receptors are major actors of leukocytes trafficking and some have been shown to play an important role in cancer metastasis. Chemokines CCL19, CCL20 and CCL21 and their receptors CCR6 and CCR7, were assessed as potential biomarkers of metastatic dissemination in primary breast cancer.

**Methods:**

Biomarker expression levels were evaluated using immunohistochemistry on paraffin-embedded tissue sections of breast cancer (n = 207).

**Results:**

CCR6 was expressed by tumor cells in 35% of cases. CCR7 was expressed by spindle shaped stromal cells in 43% of cases but not by tumor cells in this series. CCL19 was the only chemokine found expressed in a significant number of breast cancers and was expressed by both tumor cells and dendritic cells (DC). CCR6, CCL19 and CCR7 expression correlated with histologic features of aggressive disease. CCR6 expression was associated with shorter relapse-free survival (RFS) in univariate and but not in multivariate analysis (p = 0.0316 and 0.055 respectively), and was not associated with shorter overall survival (OS). Expression of CCR7 was not significantly associated with shorter RFS or OS. The presence of CCL19-expressing DC was associated with shorter RFS in univariate and multivariate analysis (p = 0.042 and 0.020 respectively) but not with shorter OS.

**Conclusion:**

These results suggest a contribution of CCR6 expression on tumor cells and CCL19-expressing DC in breast cancer dissemination. In our series, unlike what was previously published, CCR7 was exclusively expressed on stromal cells and was not associated with survival.

## Background

Most breast cancer deaths are due to metastatic relapse after treatment of apparently localized disease, and occult dissemination of tumour cells occurs at this early phase [1,2]. Post-operative systemic therapies (chemotherapy, endocrine therapy and more recently passive immunotherapy/targeted therapy with anti-HER2 monoclonal antibody) have been shown to improve recurrence-free and overall survival in patients with early breast cancer [3,4]. Molecular mechanisms of metastastic dissemination and most notably the biological mechanisms underlying organ specificities of metastatic dissemination are only partially understood [5,6]. The dissemination of breast carcinoma cell outside the organ of origin involves multiple steps, e.g. loss or deregulation of normal cell-cell contacts, production of enzymes remodelling the extracellular matrix, production of motility factors and acquisition of migratory capabilities. Several studies have reported that the chemokines/chemokine receptors system can be high jacked by epithelial tumor cells and may contribute to tumor cell dissemination [5,7-11].

Chemokine are low molecular weight proteins signalling through G-protein linked 7-transmembrane receptors and regulating the trafficking of leukocytes to lymphoid organs or inflammatory sites by inducing their migration towards the chemokine source [12]. Chemokines can be divided into inflammatory chemokines and homeostatic chemokines [13]. Chemokine and chemokine receptors have been recently identified as potential actors of the metastatic process [5,10]. Several chemokine receptors have been associated with cancer dissemination.

In the present study we sought to assess the presence of CCR6 and CCR7 receptors and their ligands in non-metastatic primary breast carcinomas. CCR6 and CCR7 were chosen because they are physiologically involved in the migration of immune cells to peripheral tissues and lymph nodes respectively. CCR6 is a chemokine receptor expressed on immature DC that binds CCL20 (also called Macrophage Inflammatory Protein (MIP) - 3α) at inflamed sites and promotes cell migration to peripheral tissues. Conversely, CCR7 is expressed on both mature DC and T lymphocytes [14,15] and binds CCL19 (also called MIP3β) and CCL21 (also called 6Ckine) chemokines which are expressed by efferent lymphatic vessels and secondary lymphoid organs, in particular lymph nodes[16]. Binding of CCR7 by its ligands promotes cell migration to secondary lymphoid organs [16].

CCR7 has been reported to be expressed on cancer cells from various origins [11,17-19], including breast cancer cells, and its expression correlates with lymph node involvement and to some extent with prognosis [7,8]. CCL20 is the sole known ligand for CCR6. CCL20 and/or CCR6 expression have been reported in pancreatic and colorectal cancer and correlates with invasion and liver metastasis [20-22]. In breast cancer, CCL20 was found to be expressed by cancer cells and correlate with infiltration by immature DC [23,24].

In the present study, we assessed the expression of CCR6, CCR7 and their ligands CCL19, CCL20 and CCL21 using immunohistochemistry in a series of tumors prospectively collected from patients with loco-regional breast cancer treated at the Centre Léon Bérard in 1996 and 1997. Results were reported in accordance with the "Reporting recommendations for tumor marker prognostic studies (REMARK)" [25]

## Methods

### Patients' selection

Clinical data from patients with early breast cancer operated at the Centre Léon Bérard were prospectively entered in a regularly updated institutional database since 1996 and paraffin embedded tumor specimen from these patients were stored. All patients with non-metastatic invasive primary breast carcinomas were eligible for this study, provided that sufficient tumor material was available for immunohistochemical analysis. One hundred and fifty six of 217 tumors met the eligibility criteria for the year 1996 and the first 100 eligible tumors (among 249 breast tumor samples) from the year 1997 were selected. Of these 256 tumor samples, those of 46 patients were excluded: 2 patients were men, 8 patients were recognized to have metastatic disease at the time of surgery, 17 patients received neoadjuvant chemotherapy (therefore the tumor sample could only be analyzed after exposure to cytotoxic chemotherapy), 16 had a previous history of invasive breast cancer (in most cases contralateral) and 3 patients had synchronous bilateral tumors. Three further tumor samples were found to be duplicates. Although 256 tumor specimens were analysed for CCR6, CCR7, CCL19, CCL20, CCL21, outcome analysis and correlation with histological and clinical parameters was therefore performed on 207 patients. Median follow up in the series for surviving patients was estimated to be 10 (range 9.8-10.1) years.

### Treatment

The following standard therapeutic procedures in the center were applied to these patients: patients with central, multiple tumors or tumors larger than 3 cm, were treated with radical mastectomy, while conservative surgery followed by radiotherapy was the standard treatment for the remaining patients; patients with nodal involvement and patients with node negative tumors and two or more adverse prognostic factors (tumor larger than 3 cm, Scarff-Bloom-Richardson (SBR) grade 3 tumors, lack of estrogen (ER) and progesterone receptor (PgR) expression, or age under 35 years) received 6 cycles of anthracyclin-containing adjuvant chemotherapy. Anthracyclin-containing adjuvant chemotherapy was also given to all patients with T4d tumors (inflammatory breast cancer); adjuvant therapy with tamoxifen 20 mg/day was given for 5 years to patients with ER and/or PgR expressing tumors. Given the timeframe of our study none of the patients received adjuvant trastuzumab.

### Immunohistochemistry

Paraffin-embedded breast tumors were serially sectioned at a thickness of 4 μm. After deparaffinization and rehydration, endogenous peroxidases were blocked by incubating the slides in 5% hydrogen peroxide in sterile water. For heat induced antigen retrieval, tissue sections were boiled in 10 mM citrate buffer pH6 using a microwave for 15 minutes [mouse anti-CCR6 clone 53103-111 (R&D Systems, Minneapolis, USA), goat polyclonal anti-CCL19/MIP3β (R&D Systems, Minneapolis, USA) antibodies], or a water bath for 40 minutes [goat polyclonal anti-CCL21-6Ckine (R&D Systems, Mineapolis, USA), and mouse anti-CCL20/MIP3α clone 308B7 (Schering-Plough, Dardilly, France) antibodies]. No antigen retrieval was performed for mouse anti-CCR7 clone 2H4 (Pharmingen, San Diego, USA) antibody.

Non-specific binding was blocked with a protein blocking reagent (Immunotech, Marseille, France) for 5 minutes except for anti-CCL19 (15 minutes). Slides were then incubated at room temperature for one hour with the primary specific antibody that was diluted using an antibody diluent solution (Chem Mate, Dako, Trappes, France) at 1/1500 for anti-CCR6, 1/25 for anti-CCL19, 1/50 for anti-CCL21, 1/200 for anti-CCL20 and 1/500 for anti-CCR7 antibodies. The primary antibody was replaced by a non immune serum for negative control slides. Slides were rinsed in phosphate buffered saline (PBS), and then incubated with a biotinylated secondary antibody bound to a streptavidin peroxidase conjugate (Ultratech HRP DAB kit, Immunotech, Marseille, France). The bound antibody was revealed by adding the substrate: 3,3'-diamino benzidine. Sections were counterstained with hematoxylin. All slides were read by a single senior pathologist (I.T.) who was blinded to the clinical data. Upon the observation of the first 30 breast cancer cases, a grading system was defined for CCR7 in which the density of positive stromal cells within the tumor was assessed semi quantitatively (4 digit system). This classification defined four groups as follows: tumors were classified as negative, or with low, intermediate or high density of positive cells as determined by the number of positive cells in at least 5 low power fields (x10) within the high staining spots. A slide which was representative for each group was then used as control for the analysis of the subsequent cases. For antibodies against CCR6 and CCL20, both the intensity of cytoplasmic staining (3 grades) and the percentage of positive tumor cells were assessed. For CCL19, both the percentage and intensity of positive tumor cells and the presence or absence of CCL19 positive DC were noted. Tumors were classified as negative, or with low, mid and high density of positive tumor cells. The few cases of discordance were reviewed by other investigators (S.G., P.A.C. or J-Y.B.) to reach a consensus.

### Statistical analysis

The correlation between the clinical and biological data and the phenotype of both tumor and stromal cells within the tumor was performed using the χ^2 ^test, Fisher exact test or non parametric tests where appropriate. For statistical analysis the semi-quantitative grading used for description of tumor samples was simplified to a 2 class system. For CCR7, tumors with low or no staining were considered negative. For CCR6 and CCL19, tumors with staining on less than 10% of tumor cells were considered negative while tumors with more than 10% of tumor cells stained were considered positive. Survival curves were plotted using the Kaplan Meier method, and were compared using the Log Rank test [26]. Multivariate analysis of prognostic factors for overall and relapse free survival were performed using a Cox model regression. No attempt was made to adjust for multiples tests. Data extraction and all statistical analysis were done using the procedures of the SPSS 12.0.1 package. This study was approved by the *Comité de Protection des Personnes SUD-EST-IV *ethics committee in Lyon

## Results

### Patients characteristics

The main characteristics of the patients are summarized in table 1. Median age was 56 (30-88) years, most tumors were 20 mm or smaller, 101 of 207 (48%) patients had node-positive tumors. Among patients with node-positive tumors, most (65 of 101, 64%) had 1 to 3 positive lymph nodes. The main histology was ductal carcinoma (83%), followed by lobular histology (11%) and approximately 5% of patients had non-ductal, non-lobular histology. One hundred and seventy four patients (84%) had hormone receptor-positive tumors (estrogen and/or progesterone receptor), 124 tumors expressed both estrogen and progesterone receptor by immunohistochemistry (IHC). HER2 was overexpressed (i.e. HER2 3+ on IHC and/or HER2 amplification on FISH) in 22 tumors (10.6%). Twenty five patients (12.1%) had triple negative breast cancer.

**Table 1:** Main characteristics of 207 patients with locoregional breast cancer

Characteristics		N	%
Total		207	100
			
Age (range)		56	(30-88)
Tumor size			
	T1	121	58,50%
	T2	76	36,70%
	T3-T4	10	4,80%
Lymph node			
	Negative	106	51,20%
	Positive	101	48,80%
Histology			
	Ductal	173	83,60%
	Lobular	24	11,60%
	Other	10	4,80%
Tumor grade (Scarff-Bloom-Richardson)			
	1	52	25,10%
	2	96	46,40%
	3	59	28,50%
Estrogen receptor			
	Negative	53	25,60%
	Positive	153	73,90%
Progesterone receptor			
	Negative	61	29,50%
	Positive	145	70,00%
			
Her2-neu expression (IHC)			
	0, 1+ or 2+	182	87,90%
	3+	22	10,60%
	Unknown	3	1,40%
CCR6 expression on Tumor cells			
	Negative	130	64,4%
	Positive	72	35,6%
CCR7 expression on Stromal cells			
	Negative	118	57,0%
	Positive	89	43,0%
CCL19 expression on Tumor cells			
	Negative	95	46,6%
	Positive	109	53,4%
CCL19 expression on Dendritic cells			
	Negative	103	50,5%
	Positive	101	49,5%

IHC: assessed by immunohistochemistry.

### Chemokines and chemokine receptors expression on tumor samples

**Figure 1:**
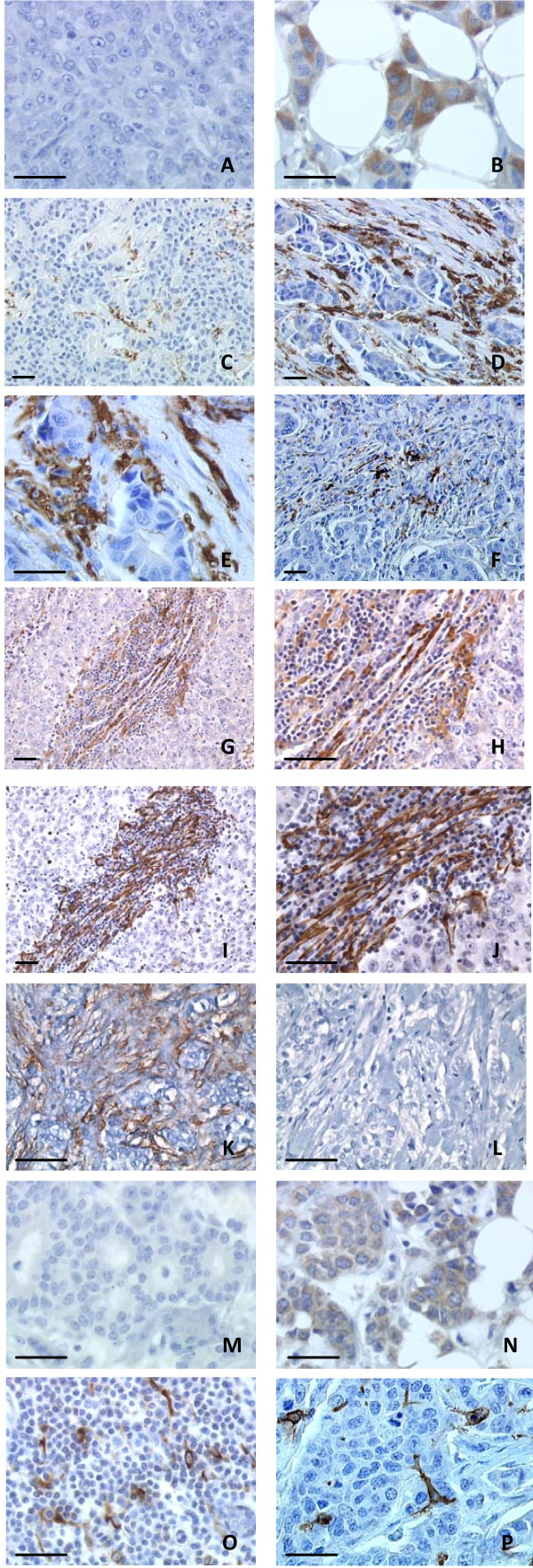
**Chemokine and chemokine receptor expression on breast tumor samples**. **Panels A & B: **CCR6 expression in breast tumors. Panel A: CCR6 negative tumor cells (× 40), and panel B: CCR6 positive tumor cells (× 40,). **Panels C, D, E & F: **CCR7 expression on tumor-infiltrating cells (× 20, panel C, × 20 panel D and × 40, panel E), panel F, CCR7+ infiltrating cells with dendritic cell morphology (× 20). **Panels G, H, I & J: **Adjacent sections (× 20 and × 40) showing elongated "fibroblastic cells" within the tumor stroma, positive for both CCR7 (panel G &H) and smooth muscle actin (SMA) (panel I & J). **Panels K & L: **staining of SMA (panel K) and caldesmone (panel L) showing lack of caldesmone staining of the SMA positive myofibroblastic cells. **Panels M & N: **CCL19/MIP3 β expression on tumor cells: panel M: negative tumor cells (× 40); panel N: positive tumor cells (× 40); **Panel O & P: **CCL19 (panel O, × 20) and CD1a (panel P, × 40) expression on cells with dendritic cell morphology in the tumor stroma. Bars are all 50 microns.

CCR6 expression was not detectable on normal breast tissue (not shown). In contrast, tumor cells expressed CCR6 in 72 samples (35%) (Figure 1A &1B and Table 1). Within the same tumor, staining was not uniform, the proportion of positive tumor cells varied from 10% to 100%. CCR6+ stromal cell were rarely detected. CCR6+, CD1a+ and Langerin+ tumor-infiltrating DC were occasionally identified in serial tissue sections from the same tumor (not shown). Although CCL20, a ligand of CCR6, was detectable in breast cell culture and frozen breast cancer tissue [24], it was not detectable in any paraffin embedded tumor samples in this series. Therefore only CCR6+ tumors cells were considered for analysis.

No CCR7+ stromal cells were detected in normal breast tissues (not shown). In contrast, 89 tumors (43%) in this series contained CCR7+ cells (Figure 1C, D, E &1F and table 1). In contrast to what was previously reported CCR7 [7,8] was not found to be expressed by tumor cells, but mostly by cells with myofibroblastic morphology within the stroma (Figure 1D). Those cells were either spread or assembled in bundles within the tumor stroma. The fibroblast-like morphology of these cells together with a detectable expression of alpha-smooth muscle actin (α-SMA) suggests that these cells might be myofibroblasts (Figure 1I &1J). Indeed these cells did not express caldesmone (Figure 1K: SMA, Figure 1L: caldesmone). In a minority of samples, a few CCR7+ cells with a dendritic morphology were observed (Figure 1F) but not retained for analysis.

CCL19, a ligand of CCR7, was expressed on tumor cells in 109 tumor samples (53%) (Figure 1M, Figure 1N and Table 1). Furthermore, CCL19-expressing with interdigited shape were found scattered in the inflammatory infiltrate in 101 tumor samples (49%)(Figure 1O &1P and Table 1). These cells also express CD1a (Figure 1P) and Langherin (additional file 1), which lead us to identify those cells as dendritic cells. CCL21 was expressed at low levels in only 9 of the first 156 patients (6%) (not shown) and for this reason its expression was not further investigated in the rest of the series.

### Association of chemokines and chemokine receptors expression with clinicopathological variables

Scarff Bloom Richardson tumor (SBR) grade was the only clinico-pathological variable associated with CCR6 expression on tumor cells (expression was associated with higher grade, p = 0.002). CCR7 expression on stromal cells was associated with lymph node involvement (but not with the number of lymph node involved), ductal histology, higher SBR grade and HER2 overexpression (p values 0.033, 0.006, 0.001 and 0.042 respectively). CCL19/MIP3β expression on tumor cells and DC had a different distribution among tumor samples were therefore analysed as two distinct biomarkers. CCL19/MIP3β expression on tumor cells was associated with higher tumor grade (p = 0.025). Tumor infiltration by CCL19/MIP3β-expressing DC was associated with both higher tumor grade (p = 0.040) and HER2 overexpression (p = 0.015). There was no significant difference in the proportions of cases with CCR6 positive tumor cells, CCR7 positive stromal cells, CCL19 positive dendritic cells, CCL19 positive tumor cells between cases classified as triple negative and cases that were not classified as such (p = 1.000; 0.392; 0.828 and 0.276 respectively).

### Association of chemokines and chemokine receptors expression with relapse-free and overall survival

**Figure 2:**
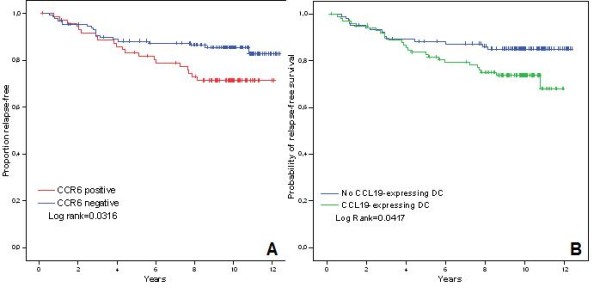
**Relapse-free survival (RFS) according to CCR6 and CCL19 expression**. **Panel A: **relapse-free survival according to CCR6 expression in tumor cells. **Panel B: **relapse-free survival according to the presence of CCL19-expressing dendritic cells (DC).

In univariate analysis, using the log rank test, larger tumor size (p = 0.0004), nodal involvement (p = 0.0036), SBR grade (p = 0.0028), lack of estrogen and progesterone receptor expression (p = 0.0123 and 0.0132 respectively), CCR6 expression (p = 0.0316)(Figure 2A) and infiltration by CCL19/MIP3β-expressing DC (p = 0.0417)(Figure 2B) were associated with shorter relapse-free survival (RFS). Interestingly, HER2 was not significantly associated with prognostic in our series, likely due to the small number of cases with HER2 overexpression (Table 2). Results of univariate analysis are summarised in Table 2. Multivariate analysis was conducted using a Cox proportional hazard model. In multivariate analysis only tumor size, node involvement and infiltration by CCL19-expressing DC were significantly associated with poorer RFS (p values of 0.017, 0.001 and 0.047 respectively)(Table 3). The association of CCR6 expression in tumor cells with shorter RFS in univariate analysis did not remain statistically significant in the multivariate analysis. None of the biomarker analysed in our study showed a significant association with overall survival (OS) neither in univariate analysis (Table 2) nor in multivariate analysis (Table 3).

**Table 2:** Summary of univariate analysis

Characteristics	N	Mean RFS (years)	95% CI	p value for LR	Mean OS (years)	95% CI	p value for LR
Age (range)							
< 40 years	15	8.1	6.3-10.0		10.6	10.1-11.1	
40-55 years	82	11.1	10.5-11.7		11.2	10.7-11.8	
> 55 years	110	10.3	9.6-11.1	0.084	10.2	9.5-10.9	0.051
Tumor size							
T1	121	11.1	10.6-11.7		11.1	10.6-11.7	
T2	76	10.0	9.1-10.8		10.5	9.9-11.2	
T3-T4	10	6.1	2.9-9.4	**< 0.001**	6.6	3.6-9.6	**< 0.001**
Lymph node							
Negative	106	11.0	10.5-11.7		11.2	10.7-11.7	
Positive	101	10.0	9.2-10.7	**0.031**	10.1	9.4-10.8	**0.030**
Histology							
Ductal	173	10.6	10.0-11.1		10.6	10.1-11.1	
Lobular	24	10.5	9.1-11.9		11.5	10.8-12.3	
Other	10	9.8	7.4-12.3	0.722	10.4	7.8-12.9	0.410
Tumor grade (Scarff-Bloom-Richardson)							
I	52	11.9	11.4-14.4		11.7	11.1-12.3	
II	96	10.4	9.6-11.1		10.6	10.0-11.2	
III	59	9.4	8.2-11.1	**0.003**	9.9	8.9-10.9	**0.024**
Estrogen receptor							
Negative	53	9.6	8.4-10.7		10.2	9.2-11.2	
Positive	153	11.0	10.5-11.5	**0.012**	11.0	10.5-11.5	0.354
Progesterone receptor							
Negative	61	9.5	8.4-10.6		9.9	9.0-10.9	
Positive	145	11.1	10.6-11.6	**0.013**	11.2	10.7-11.6	**0.035**
Her2-neu expression (IHC)							
0, 1+ or 2+	182	10.6	10.1-11.1		10.7	10.3-11.2	
3+	22	9.7	8.4-10.9	0.819	10.1	9.2-11.0	0.886
CCR6 on tumor cells							
Negative	130	11.0	10.4-11.6		10.9	10.3-11.4	
Positive	72	9.9	9.0-10.8	**0.032**	10.5	9.8-11.2	0.400
CCR7 on stromal cells							
Negative	118	10.8	10.2-11.5		11.0	10.5-11.6	
Positive	89	10.3	9.4-11.1	0.344	10.4	9.6-11.1	0.153
CCL19 on tumor cells							
Negative	95	10.4	9.6-11.2		10.7	10.0-11.3	
Positive	109	10.7	10.0-11.4	0.730	10.8	10.2-11.4	0.962
CCL19 on dendritic cells							
Negative	103	11.0	10.4-11.7		11.0	10.3-11.5	
Positive	101	9.8	9.1-10.6	**0.042**	10.2	9.6-10.9	0.271

IHC: assessed by immunohistochemistry.

**Table 3:** Summary of multivariate analysis.

Relapse-free survival			
Variable	Hazard Ratio	95% CI	p value
Tumor size (mm)	1.031	[1.005-1.056]	**0.018**
Number of positive node	1.086	[1.030-1.155]	**0.002**
SBR grade 2 (ref. SBR1)	1.945	[0.551-6.871]	0.302
SBR grade 3 (ref. SBR1)	2.349	[0.628-8.794]	0.205
Estrogen receptor (negative)	1.527	[0.658-3.543]	0.325
Progesterone receptor (negative)	1.424	[0.6593-3.102]	0.374
CCR6+ tumor cells	1.929	[0.986-3.772]	0.055
CCL19+ DC	2.277	[1.141-3.772]	**0.020**

**Overall Survival**			
**Variable**	**Hazard Ratio**	**95% CI**	**p value**

Tumor size (mm)	1.027	[1.006-1.049]	**0.012**
Number of positive node	1.118	[1.066-1.173]	**< 0.001**
SBR grade 2 (ref. SBR1)	1.534	[0.549-4.287]	0.415
SBR grade 3 (ref. SBR1)	1.931	[0.666-5.605]	0.226
Progesterone receptor (negative)	1.419	[0.751-2.678]	0.281
CCR6+ tumor cells	1.205	[0.638-2.277]	0.565
CCL19+ DC	1.717	[0.916-3.219]	0.092

SBR: Scarff-Bloom-Richardson grade; ref.: reference for analysis; CCR6+: CCR6-positive; CCR19+ DC: CCR19-positive dendritic cells.

### CCR7-stromal cells are found in metastatic axillary lymph nodes

**Figure 3:**
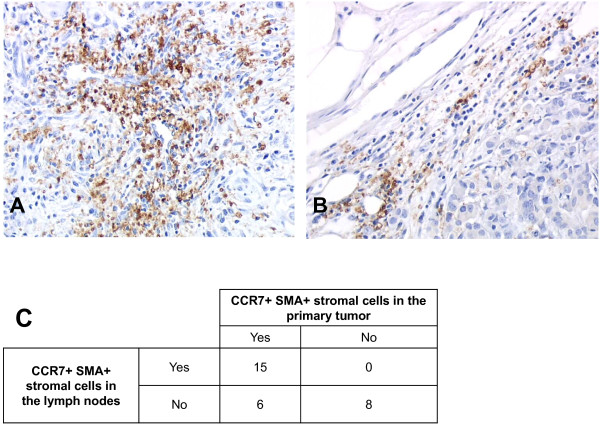
**CCR7-expressing stromal cells infiltrate metastatic lymph nodes**. **Panels A & B: **CCR7+ myofibroblasts in axillary lymph nodes (LN) of node positive tumors (panel A) and CCR7+ myofibroblasts in an invaded lymph node with capsular invasion (panel B). **Panel C: **Number of patients with CCR7+stromal cells among patients with positive lymph nodes (for tumor invasion) according to the presence of CCR7+ stromal cells in the primary tumor, p = 0.001 (Fisher' test).

Since CCR7 is involved in the migration of DC and T cells from peripheral tissues to the draining axillary lymph nodes, the presence of CCR7-expressing stromal cells was investigated in axillary lymph nodes (LN) (Figure 3A &3B). The lymph nodes and primary tumor of 49 randomly selected patients were examined, 34 patients had lymph node invasion and 15 had no lymph node invasion. A total of 184 lymph nodes were analysed, 85 of them were invaded by tumor cells (18 micro-metastases and 67 macro-metastases). Among the patients with lymph node positive tumors, 3 LN from 2 patients with micro-metastases could not be analysed because of insufficient material left, while for 3 other patients, CCR7 expression could not be assessed in the primary tumor. No CCR7+/α-SMA+cells could be observed in normal LN (i.e. without tumor cells) whether from node-negative or node positive axillary LN dissection (not shown). However, a few CCR7+/α-SMA- cells with dendritic morphology were scattered among T cells and were identified as mature interdigitating DC. In contrast, CCR7+/α-SMA+ stromal cells were observed in 34 of 85 tumor-invaded LN, most often in the "histiocytic" areas of the lymph node, in or beneath the sub-capsular sinus (Figure 3A). All CCR7-expressing stromal cells were observed in the invaded axillary lymph nodes of 15 of the 21 patients whose primary tumors contained CCR7-expressing stromal cells versus 0 of the 8 patients with CCR7-negative tumors (p = 0.001)(Figure 3C). CCR7-expressing stromal cells had the same morphology and distribution in the primary tumor and in the invaded lymph nodes (Additional file 2). Of note, in 3 of 3 lymph nodes where metastatic cells had eroded the LN capsule, CCR7+/α-SMA+ stromal cells were observed at the front line of capsular invasion (Figure 3B). In the regional axillary lymph nodes, CCR7+ myofibroblasts are therefore observed only in case of nodal involvement by tumor cells and only from primary tumors containing CCR7-expressing stromal cells.

## Discussion

The dissemination of breast carcinoma cell outside the organ of origin involves multiple steps, e.g. loss or deregulation of normal cell-cell contacts, production of enzyme remodelling the extracellular matrix, production of motility factors and acquisition of migration capacities. Several studies have reported that chemokines and their receptors can be expressed by epithelial tumor cells and may contribute to cell migration [5,7-11].

The objective of the present study was to investigate the presence of CCR6 and CCR7 chemokine receptors and their ligands in non-metastatic primary breast carcinomas. CCR6 and CCR7 were chosen because they are physiologically involved in the migration of immune cells to peripheral tissues and lymph nodes respectively. Expression of these chemokine and chemokine receptors was assessed using IHC. CCR6, CCR7 and CCL19 were found to be expressed in a large proportion of tumors and were, in most cases, associated with features of more aggressive disease, such as higher histological grade and HER2 overexpression. We could not detect any CCL20 expression in our series using IHC on paraffin embedded tissue sample while we were previously able to show CCL20 expression on frozen tissue section [24], using a different antibody. Lack of CCL20 detection in our series may therefore be regarded as a technical limitation. CCL21 was expressed at low levels in very few tumors in this series. Further studies, using different techniques such as quantitative RT-PCR or protein extraction on frozen samples, will be needed to accurately assess the expression of CCL20 and CCL21 in primary breast cancer. CCR6 was detectable in tumor cells, while CCL19 was expressed by both tumor cells and stroma-infiltrating cells exhibiting a dendritic morphology. In this series CCR7 was found to be expressed only on stromal cells either with myofibroblastic morphology and α-SMA expression or with dendritic morphology, but not on tumor cells. In previously reported series, CCR7 expression had been described on tumor cells from breast cancer [7,8] as well as from other tumors such as gastric and lung cancer [11,19] but not on cells in the tumor stroma. The reasons for this discrepancy are not clear, and may possibly reflect different methods used for IHC staining. Indeed, André et al [7] and Cabioglu et al [8] both used the same anti-CCR7 antibody, but with different peroxydase bloking agents and different antibody incubation time. (Cabioglu et al 2005 [8] and F. André, personal communication). The precise nature of these CCR7-expressing cells remains unclear. Despite the fact that CCR7 was found on stromal cells in the present study this feature was still significantly associated with node involvement. However, unlike what was previously reported and despite the fact the CCR7-expression on stromal cells was associated with several features of aggressive disease, we were unable to show any correlation between CCR7 expression and poorer outcome whether in univariate or multivariate analysis. This finding also raises concerns on the consistency and interpretation of IHC studies and therefore on the role of IHC in the identification of biomarkers.

CCR6 was expressed on tumor cells and its expression was associated with shorter relapse-free survival in univariate but not in multivariate analysis, and its impact on overall survival was not statistically significant. Although CCR6 expression has been reported in several cancers and usually correlates with more aggressive disease [20,21,27], it's association with shorter RFS has never been demonstrated before. CCR6 may increase tumor cells' motility and therefore their metastatic potential by acting on the cytoskeleton, as has been described in a model of colonic epithelium [28].

We also found that tumor infiltration by CCL19-expressing DC was associated with shorter relapse-free survival in both univariate and multivariate analysis. However since mature DCs are known to express both CCR7 and CCL19 [29] the patho-physiological meaning of this finding is unclear. Indeed since we did not use other markers of DC, this finding may reflect the fact that tumor infiltration by DC is of poor prognosis as suggested in a previous report[30].

Finally, we found that CCR7-expressing stromal cells were also present in lymph nodes invaded by tumor cells but this finding was restricted to the patients who had CCR7-expressing stromal cells in their primary tumor. This finding suggests that tumor cells may be able to recruit CCR7-expressing stromal cells.

## Conclusion

Our results suggest that CCR6 expression on tumor cells and that infiltration by CCL19-expressing DC contributes to breast cancer dissemination. In our series, unlike what was previously published, CCR7 was exclusively expressed on stromal cells and was not associated with survival.

## Competing interests

The authors declare that they have no competing interests.

## Authors' contributions

PAC analysed the data and drafted the manuscript, IT designed the study, performed the histological analysis, supervised the immunostaining, analysed the data, and drafted the manuscript, TB designed the study, provided study material and helped draft the manuscript; IRC provided study material; NBV participated in study design and data analysis and helped draft the manuscript; CMC participated in study design and data analysis and helped draft the manuscript, OT provided study material, SG performed the immunostaining and analysed the data, JJP participated in study design, HM provided study material, CBD participated in study design and data analysis, CC participated in study design and data analysis and helped draft the manuscript, SL conceived and designed the study, JYB conceived and designed the study, analysed the data and drafted the manuscript. All authors have read and approved the final manuscript.

## Pre-publication history

The pre-publication history for this paper can be accessed here:

http://www.biomedcentral.com/1471-2407/11/213/prepub

## Supplementary Material

Additional file 1 **Langherin expression on dendritic cells in the stroma of breast cancer**. Panel A ×20 and Panel B ×40. Click here for file

Additional file 2 **CCR7-expressing stromal cells display the same morphology and distribution pattern in primary tumor and matching invaded lymph nodes**. Panels A-D, Case 1: lymph node × 20 (panel A), lymph node × 40 (panel B), primary tumor × 20 (panel C), primary tumor × 40 (panel D). Panels E-H, case 2: lymph node × 20 (panel E), lymph node × 40 (panel F), primary tumor × 20 (panel G), primary tumor × 40 (panel H). Click here for file
