# Supplementary figures and images for: Prognostic value of the expression of C-Chemokine Receptor 6 and 7 and their ligands in non-metastatic breast cancer
Source: BMC Cancer. 2011 May 30;11:213. doi: 10.1186/1471-2407-11-213 (PMC3130701; doi:10.1186/1471-2407-11-213)

## Slide 1
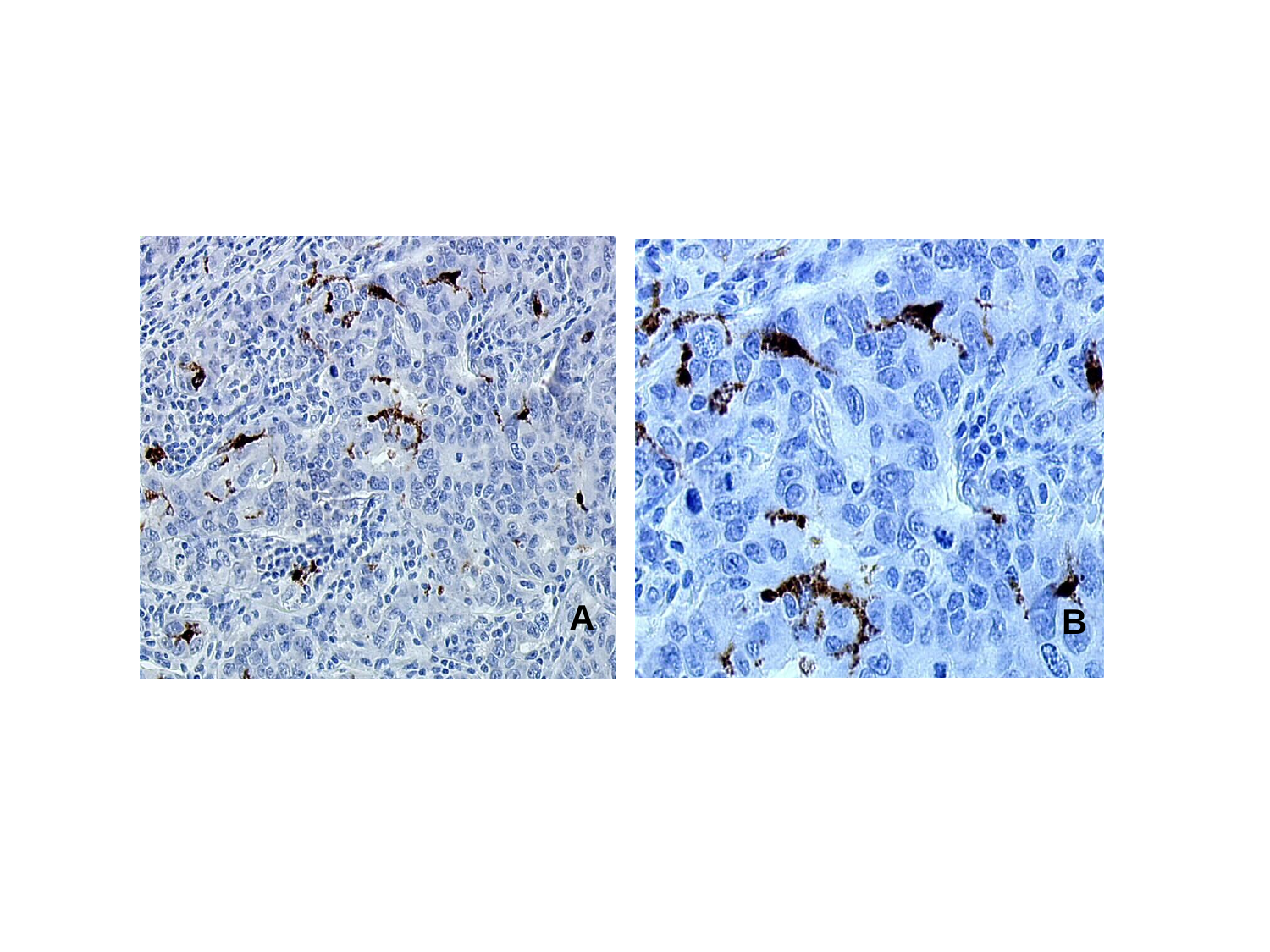

A
B

Supplement: Additional file 1 — Langherin expression on dendritic cells in the stroma of breast cancer. Panel A ×20 and Panel B ×40. [file 1471-2407-11-213-S1.PPT]

## Slide 1
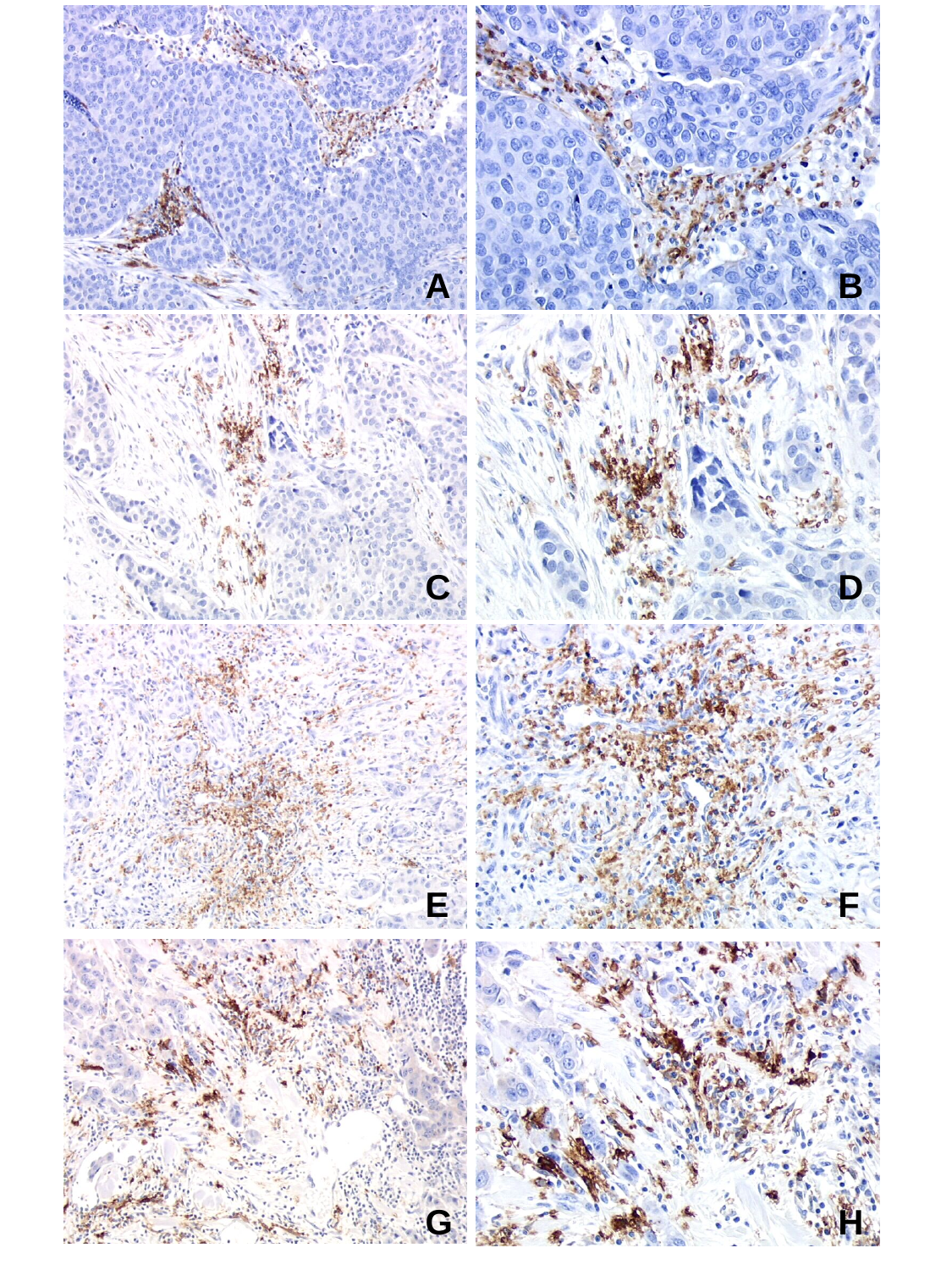

Supplement: Additional file 2 — CCR7-expressing stromal cells display the same morphology and distribution pattern in primary tumor and matching invaded lymph nodes. Panels A-D, Case 1: lymph node × 20 (panel A), lymph node × 40 (panel B), primary tumor × 20 (panel C), primary tumor × 40 (panel D). Panels E-H, case 2: lymph node × 20 (panel E), lymph node × 40 (panel F), primary tumor × 20 (panel G), primary tumor × 40 (panel H). [file 1471-2407-11-213-S2.PPT]
